# Axitinib after Treatment Failure with Sunitinib or Cytokines in Advanced Renal Cell Carcinoma—Systematic Literature Review of Clinical and Real-World Evidence

**DOI:** 10.3390/cancers16152706

**Published:** 2024-07-30

**Authors:** Anand Sharma, Amit Bahl, Ricky Frazer, Esha Godhania, Nicholas Halfpenny, Kristina Hartl, Dorothea Heldt, John McGrane, Sera Şahbaz Gülser, Balaji Venugopal, Aimi Ritchie, Katherine Crichton

**Affiliations:** 1 Mount Vernon Cancer Centre, Northwood HA6 2RN, UK; 2 University Hospitals Bristol & Weston NHS Trust, Bristol BS2 8ED, UK; 3 Velindre Cancer Centre, Cardiff CF14 2TL, UK; 4 Pfizer Ltd., Walton Oaks, Tadworth KT20 7NS, UK; 5 Open Health Group, 3068 AV Rotterdam, The Netherlands; 6 Open Health Group, 10117 Berlin, Germany; 7 Royal Cornwall Hospitals NHS Trust (Treliske), Truro TR1 3LJ, UK; 8 Beatson West of Scotland Cancer Centre, Glasgow G12 0YN, UK

**Keywords:** renal cell carcinoma, axitinib, systematic literature review

## Abstract

**Simple Summary:**

Roughly 430,000 new cases of renal cell carcinoma (RCC) occurred worldwide in 2022, and about one-third of RCC cases are diagnosed at an advanced stage of disease (aRCC). When patients with aRCC fail first-line treatment, they commonly receive one of the recommended second-line treatments, i.e., axitinib, cabozantinib, lenvatinib plus everolimus, pazopanib, sunitinib, or tivozanib. Axitinib is a second-generation tyrosine kinase inhibitor that inhibits vascular endothelial growth factor receptor tyrosine kinases 1, 2, and 3. It has been available since 2011 for the treatment of aRCC after the failure of prior therapy with sunitinib or cytokines. The aim of our research was to understand how axitinib compares with other treatment options considering data from clinical trials and observational studies that reflect real-world clinical practice. Therefore, we conducted a systematic literature review to summarise evidence on commonly used and recommended treatments in aRCC after the failure of prior therapy with sunitinib or cytokines.

**Abstract:**

Background: We conducted a systematic literature review (SLR) to identify clinical evidence on treatments in advanced renal cell carcinoma (aRCC) after the failure of prior therapy with cytokines, tyrosine kinase inhibitors, or immune checkpoint inhibitors. Herein, we summarise the evidence for axitinib in aRCC after the failure of prior therapy with cytokines or sunitinib. Methods: This SLR was registered with PROSPERO (CRD42023492931) and followed the 2020 PRISMA statement and the Cochrane guidelines. Comprehensive searches were conducted in MEDLINE and Embase as well as for conference proceedings. Study eligibility was defined according to population, intervention, comparator, outcome, and study design. Results: Of 1252 titles/abstracts screened, 266 peer-reviewed publications were reviewed, of which 182 were included. In addition, 28 conference abstracts were eligible. Data on axitinib were reported in 55 publications, of which 16 provided efficacy and/or safety outcomes on axitinib after therapy with sunitinib or cytokines. In these patients, median progression-free and overall survival ranged between 5.5 and 8.7 months and 11.0 and 69.5 months, respectively. Conclusions: Axitinib is commonly used in clinical practice and has a well-characterised safety and efficacy profile in the treatment of patients with aRCC after the failure of prior therapy with sunitinib or cytokines.

## 1. Introduction

Renal cell carcinoma (RCC) had a global incidence of roughly 434,000 cases in 2022 [1]. Approximately one-third of all RCC cases are diagnosed at an advanced stage with metastatic disease [2]. For advanced renal cell carcinoma (aRCC), cytokines, such as interleukin-2 or interferon-alpha, were the standard first-line treatment for many years until the advent of tyrosine kinase inhibitors (TKIs) [3,4,5]. Sunitinib was the first TKI approved by the European Medicines Agency in 2006, followed by several other TKIs and mammalian target of rapamycin (mTOR) inhibitors [3]. Between 2009 and 2019, TKIs and mTOR inhibitors were recommended by the European Society for Medical Oncology (ESMO) as the first-line treatment for aRCC [3]. Since 2019, immune checkpoint inhibitors (ICIs) have been available for the first-line treatment of aRCC, and ESMO now recommends the combination of ICI with TKI as a first-line treatment for aRCC [3,6]. In patients with a favourable risk profile according to the International Metastatic RCC Database Consortium (IMDC), ESMO recommends first-line treatment with lenvatinib plus pembrolizumab, axitinib plus pembrolizumab, or cabozantinib plus nivolumab [6]. In patients with intermediate- or poor-risk disease, the ICI combination of ipilimumab plus nivolumab is another recommended first-line option for patients with aRCC in addition to the combination therapies recommended for favourable-risk groups [6].

In the subsequent second-line setting, ESMO recommends the use of either axitinib, cabozantinib, lenvatinib plus everolimus, pazopanib, sunitinib, or tivozanib, depending on which prior treatment patients with aRCC have received and irrespective of the IMDC risk classification of their disease [6].

Axitinib is a potent and highly selective second-generation TKI, inhibiting vascular endothelial growth factor receptor tyrosine kinases 1, 2, and 3 [7,8]. The medicine has been available since 2011 for the second-line treatment of aRCC after the failure of prior therapy with either cytokines or sunitinib. Whilst the recommended dose is 5 mg twice daily, dosing is flexible with axitinib and can be increased up to 10 mg twice daily or reduced as needed for the individual patient [8]. The half-life of axitinib is short at 2.5 to 6.1 h [7].

As axitinib has been on the market for more than ten years and many other agents have become available in the meantime for the treatment of aRCC, it is important to understand how axitinib currently compares with other treatment options considering the data from clinical trials and observational studies reflecting clinical practice. Therefore, we conducted a systematic literature review (SLR) to identify the clinical evidence on commonly used and recommended treatments in aRCC after the failure of prior therapy with cytokines, TKIs or ICIs. In this paper, we summarise the available evidence for axitinib after the failure of prior therapy with cytokines or sunitinib, in accordance with its label.

## 2. Methods

Methods employed in this SLR followed the 2020 Preferred Reporting Items for Systematic Reviews and Meta-Analyses (PRISMA) statement and the Cochrane guidelines and were documented in a study protocol [9,10]. The SLR was registered with PROSPERO (CRD42023492931) [11].

The scope of this literature review was defined according to population, intervention, comparators, outcomes, and study design (PICOS) criteria. Studies of interest for this SLR included randomised controlled trials (RCTs), non-randomised trials, and observational studies that investigated the efficacy and safety of treatments in patients with aRCC who had failed prior treatment with cytokines, TKI monotherapy, ICI monotherapy, TKI-ICI combinations, or ICI combinations. To be included in the review, a study had to fulfil each of the PICOS criteria as outlined in detail in Table 1.

A comprehensive set of search terms was developed to search the MEDLINE and Embase databases via the ProQuest search engine on 6 November 2023. In addition, conference abstracts for the years 2021, 2022, and 2023 from the American Society of Clinical Oncology (ASCO) [12], ASCO Genitourinary (ASCO GU) [12], American Urological Association (AUA) [13], European Association of Urology (EAU) [14], ESMO [15], International Kidney Cancer Symposium (IKCS) [16] and European International Kidney Cancer Symposium (EIKCS) [16], and the Society of Immunotherapy of Cancer (SITC) [17] were searched. The results of all literature searches were downloaded and imported into an EndNote database where duplicates were removed. Based on the predefined eligibility criteria (Table 1), the screening of all references was performed by two independent reviewers using the literature review software DistillerSR (version 2.35). A third reviewer was consulted as needed to resolve any decision discrepancies between the two reviewers. Data were extracted from the final set of included articles. Data extraction was performed by a single reviewer and a second reviewer independently checked the accuracy of all extracted variables against a clean copy of the publication.

Study quality was assessed using the checklists from the Centre for Reviews and Dissemination (CRD) Guidance for Undertaking Reviews in Health Care (2009) [18] and the checklist from Downs and Black (1998) [19]. The checklist by Downs and Black was based on the original version; however, question 27 concerning power was modified, in line with previously published research [20,21,22,23]. The question was answered with yes (score of 1) if the authors reported performing a power analysis. In any other case, the question was answered with no or unable to determine (score of 0). The maximum score of the modified checklist was 28. The risk of bias assessment was conducted at the study level by two blinded reviewers and adjudicated by a third independent reviewer in case of any discrepancies.

## 3. Results

### 3.1. Literature Search and PRISMA

**Figure 1:**
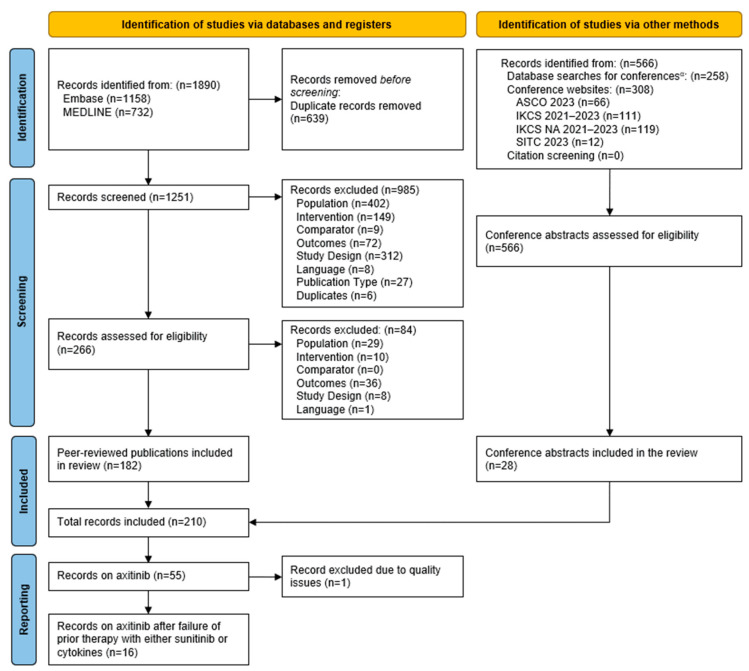
PRISMA flow diagram. ^α^ Conferences covered in Embase database search (2021–2023): American Society of Clinical Oncology, ASCO Genitourinary, American Urological Association, European Association of Urology, European Society for Medical Oncology, International Kidney Cancer Symposium, European International Kidney Cancer Symposium, Society of Immunotherapy of Cancer.

The database searches in Embase and MEDLINE identified a total of 1890 records. After the removal of duplicates (n = 639), 1251 titles/abstracts were screened for eligibility according to the predefined PICOS criteria. Subsequently, a total of 266 full-text articles were screened, which resulted in the inclusion of 182 peer-reviewed publications for data extraction. In addition, 28 abstracts from the conference proceedings searches met the inclusion criteria. Of the included records, 55 provided data on axitinib. Of these, one record was excluded from reporting as the quality of the publication was judged insufficient by the reviewers. Eventually, a total of 16 records provided efficacy and/or safety outcomes on axitinib after the failure of prior therapy with either sunitinib or cytokines. The PRISMA diagram of this SLR is provided in Figure 1.

### 3.2. Study and Patient Characteristics

In this publication, we focus solely on the available evidence for axitinib after the failure of prior therapy with cytokines and/or sunitinib, according to its label [8]. We report efficacy outcomes in terms of progression-free survival (PFS), overall survival (OS), and objective response rate (ORR), safety outcomes in terms of discontinuation due to adverse events (AE), and health-related quality of life (HRQoL) outcomes.

#### 3.2.1. RCTs and Single-Arm Clinical Trials

A total of six publications reported clinical evidence from RCTs of axitinib after prior cytokine or sunitinib regimens (Table 2). Five of these publications reported data from the open-label phase 3 clinical trial AXIS (NCT00678392), in which axitinib was compared with sorafenib in patients with aRCC who had failed first-line therapy with either sunitinib, cytokines, bevacizumab plus interferon-alpha, or temsirolimus [24,25,26,27,28]. The AXIS trial was a multicentre, international RCT including a total of 723 patients with clear-cell aRCC, of whom 361 and 362 were randomly assigned to axitinib and sorafenib, respectively [25]. The primary publication of the AXIS trial by Rini et al. (2011) reported on PFS outcomes, response rates, AEs, dose intensity, and HRQoL outcomes [24]. Updated results including OS data were provided by Motzer et al. (2013) [28]. The publication by Cella et al. (2013) [26] focused specifically on patient-reported outcomes, Ueda et al. (2013) [27] focused on the subgroup outcomes of Japanese patients, and Bracarda et al. (2019) [25] published prognostic factor analyses from the AXIS trial. Baseline characteristics for the subgroup of patients who received prior sunitinib or cytokines were only available for a subset of Japanese patients and therefore are not reported herein [27].

The ESCAPE study (Kadono et al., 2023), an open-label phase 3 RCT, compared the efficacy of axitinib based on the prior treatment regimen, i.e., first-line cytokines (interleukin-2 plus interferon-alpha) versus first-line sunitinib [29]. The included patients had clear-cell mRCC with a favourable or intermediate risk profile. The ESCAPE study was conducted in Japan and randomised 35 patients to receive either treatment with cytokines or sunitinib [29]. After the discontinuation of first-line treatment, a total of 18 patients received axitinib; of these, 13 and 5 patients had prior cytokines (54.5%) and sunitinib (45.5%), respectively (Supplementary Table S1) [29].

Three publications reported on single-arm clinical trials assessing the efficacy and safety of axitinib (Table 2) [20,21,22,23,24,25,26,27,28,29,30,31,32]. The publication by Rixe et al. (2007) reported on a phase 2 study of axitinib including a total of 52 patients with metastatic RCC (mRCC) who failed prior therapy with cytokines [32]. Prior cytokine treatments included interferon-alpha alone (52%), interleukin-2 alone (17%), or both combined (15%). The median (range) age of the patients was 59 years (35–85), and 23% of the patients were female. Furthermore, 60% of the patients had an ECOG PS of 0, while the remaining patients (40%) had a score of 1. The risk profile was favourable in 42% of the patients and intermediate or poor in 52% of the patients. Aside from 2% of patients with papillary cell RCC, all other patients had clear-cell RCC (ccRCC). The majority of the patients (94%) had undergone prior nephrectomy (Table S1) [32]. Eto et al. (2014) reported on a phase 2 study of axitinib after the failure of prior therapy with cytokines [30]. This Japanese single-arm clinical trial included 64 patients with mRCC with a clear-cell component [30]. All patients received first-line treatments with either interferon-alpha or interleukin-2 [30]. The median (range) age of the patients was 63 years (34–80), and approximately one-third of the patients were female. In addition, most patients were relatively fit with an ECOG score of 0 (89%) and showed a favourable (17%) to intermediate (77%) risk profile based on the Memorial Sloan-Kettering Cancer Center (MSKCC) score [30]. Lastly, almost all patients had ccRCC (97%), and 2% of the patients had papillary cell RCC (Table S1) [30]. In the phase 2 study by Rini et al. (2009), patients received third-line treatment with axitinib after the failure of sorafenib and sunitinib or cytokines [31]. The trial included a total of 62 patients with mRCC [31].

#### 3.2.2. Observational Studies

A total of six observational studies were identified that reported on the outcomes of axitinib treatment after the failure of prior therapy with sunitinib or cytokines (Table 2) [33,34,35,36,37,38]. The study by Cesas et al. (2023) conducted in Lithuania included retrospective and prospective cohorts [33]. The 143 patients with mRCC who participated in this study received sunitinib or pazopanib as first-line treatment and either axitinib, cabozantinib, everolimus, or nivolumab as second-line treatment [33]. Among the patients who received sunitinib as first-line treatment, the proportion of patients with an IMDC favourable risk score was highest in the axitinib group (32.7%), followed by the cabozantinib group (16%) and the nivolumab group (13.5%) [33]. The rates for intermediate- and poor-risk groups were 57.7% and 9.6% in the axitinib cohort, 80% and 4% in the cabozantinib cohort, and 67.6% and 18.9% in the nivolumab cohort, respectively (Table S1) [33].

**Table 2:** Characteristics of included studies.

Author, Year	Country	Study Design	Study Population (N)	Tx (n)	Tx Line	Prior Tx	Outcomes
Randomised controlled trials							
Rini, 2011 [24]; Bracarda, 2019 [25]; Cella, 2013 [26]; Motzer, 2013 [28]; Ueda, 2013 [27] AXIS (NCT00678392)	International	Phase 3, open-label	Patients with aRCC who had disease progression after initial systemic therapy (N = 723)	I: Axitinib (n = 361) C: Sorafenib (n = 362)	2L	1L: Sunitinib (n = 389) Cytokines (n = 251) Bevacizumab (n = 59) Temsirolimus (n = 24)	Primary outcome: PFS Secondary outcomes: OS, ORR, duration of response, TTD, safety, patient-reported outcomes
Kadono, 2023 [29] ESCAPE (UMIN000012522)	Japan	Phase 3, open-label	Patients with favourable and intermediate risk mRCC (N = 35)	I: Axitinib after cytokines (n = 35) C: Axitinib after sunitinib (n = 15)	2L	1L: Cytokines (n = 18) Sunitinib (n = 15)	Primary outcome: PFS Secondary outcomes: OS, ORR, DCR, safety
Single-arm clinical trials							
Eto, 2014 [30] (NCT00569946)	Japan	Phase 2, single-arm	Patients with mRCC with a clear-cell component (N = 64)	Axitinib (n = 64)	≥2L	1L or 2L: Cytokines (n = 64)	Primary outcome: ORR Secondary outcomes: OS, PFS, duration of response, safety, pharmacokinetics
Rini, 2009 [31] (NCT00282048)	US	Phase 2, single-arm	Patients with refractory mRCC (N = 62)	Axitinib (n = 62)	≥2L	1L or 2L: Sorafenib (n = 62) Sunitinib (n = 14) Cytokines (n = 38) Chemotherapy (n = 12) Bevacizumab (n = 5) Temsirolimus (n = 3)	Primary outcome: ORR Secondary outcomes: OS, PFS, duration of response, safety, patient-reported outcomes, pharmacokinetics
Rixe, 2007 [32] (NCT00076011)	France, Germany, US	Phase 2, single-arm	Patients with mRCC who had failed previous cytokine-based treatment (N = 52)	Axitinib (n = 52)	2L	1L: Cytokines (n = 52)	Primary outcome: ORR Secondary outcomes: OS, PFS, duration of response, time to progression, safety, pharmacokinetics, HRQoL
Observational studies							
Cesas, 2023 [33]	Lithuania	Retrospective and prospective study cohorts	Patients with mRCC who had received 1L VEGF-targeted therapy with either sunitinib or pazopanib (N = 143)	Axitinib (n = 59) Cabozantinib (n = 30) Everolimus (n = 8) Nivolumab (n = 46)	≥2L	1L: Sunitinib (n = 123) Pazopanib (n = 20)	Outcomes: PFS2
Facchini, 2019 [34]; D’Aniello, 2016 [39]	Italy	Retrospective	Patients with mRCC (N = 148)	Axitinib (n = 148)	2L	1L: Sunitinib (n = 148)	Primary outcomes: PFS, OS, ORR, DCR, safety Secondary outcomes: relationship between patients’ demographic and baseline characteristics, AEs, and response
Géczi, 2020 [35]	Hungary	Retrospective	Patients with mRCC (N = 512)	Axitinib (n = 128) Everolimus (n = 384)	2L	1L: Sunitinib (n = 446) Pazopanib (n = 66)	Outcomes: OS, duration of 1L treatment
Iacovelli, 2018 [36]	Italy	Retrospective	Patients with metastatic ccRCC (N = 182)	Axitinib (n = 103) Everolimus (n = 79)	2L	1L: Sunitinib (n = 182)	Outcomes: PFS, OS
Kang, 2023 [37]	Korea	Retrospective	Patients with mRCC (N = 3247)	Axitinib (n = 773) Everolimus (n = 2198) Cabozantinib (n = 276)	2L	1L: Sunitinib (n = 1787) Pazopanib (n = 1460)	Outcome: OS
Tamada, 2018 [38]	Japan	Retrospective	Patients with mRCC (N = 83)	Axitinib (n = 52) Everolimus or temsirolimus (n = 31)	2L	1L: Sunitinib (n = 83)	Outcomes: OS, PFS, time to treatment failure

1L, first-line; 2L, second-line; AE, adverse event; aRCC, advanced renal cell carcinoma; c, comparator; ccRCC, clear-cell renal cell carcinoma; DCR, disease control rate; DoR, duration of response; DoT, duration of treatment; HRQoL, health-related quality of life; I, intervention; mRCC, metastatic renal cell carcinoma; N, number of study population; n, number of patients; ORR, overall response rate; OS, overall survival; PFS, progression-free survival; PK, pharmacokinetics; RCC, renal cell carcinoma; TTD, time to treatment discontinuation; tx, treatment; VEGF, vascular endothelial growth factor.

D’Aniello et al. (2016) [39] and Facchini et al. (2019) [34] reported on the SAX study conducted in Italy. In this retrospective study, all 148 patients with mRCC received axitinib after the failure of first-line sunitinib [34,39]. The median (range) age of the patients was 62 years (35–85), and 49.3% of the patients were female [34]. Approximately half of the patients had an ECOG PS of 0 (55.4%), followed by a score of 1 (41.3%) and 2 (3.3%) [34]. The MSKCC and IMDC risk classifications were comparable at 27.7% and 24.3% in the favourable group, 60.8% and 60.1% in the intermediate group, and 11.5% and 15.5% in the poor group, respectively [34]. Most of the patients had ccRCC (94%) and had undergone prior nephrectomy (90.5%) (Table S1) [34]. Another Italian retrospective observational study by Iacovelli et al. (2018) included 182 mRCC patients who received first-line treatment with sunitinib followed by second-line treatment with either axitinib or everolimus [36]. The median age of the patients was 58.3 years in the axitinib group and 60.6 years in the everolimus group [36]. The proportion of female patients was 19.4% and 38%, respectively [36]. The IMDC risk groups were comparable between axitinib and everolimus cohorts at 18% and 21.7% in the favourable-risk groups, 67% and 65.8% in the intermediate-risk groups, and 15% and 12.7% in the poor-risk groups, respectively [36]. All patients had ccRCC, and 89.3% of the patients in the axitinib group and 91% of the patients in the everolimus group had undergone prior nephrectomy (Table S1) [36].

The retrospective claims database study by Géczi et al. (2020) was conducted in Hungary and included 512 patients with mRCC [35]. In this study, patients received either first-line sunitinib followed by second-line axitinib, sunitinib followed by everolimus, or pazopanib followed by everolimus [35]. The mean age of the patients who received prior treatment with sunitinib was 62.1 years in the axitinib group and 60.9 years in the everolimus group (Table S1) [35].

Kang et al. (2023) conducted a retrospective database study in Korean patients with mRCC (N = 3247) who were treated with first-line sunitinib or pazopanib followed by second-line treatment with either axitinib, cabozantinib, or everolimus [37]. The study authors reported efficacy outcomes for the subgroup of patients who received sunitinib as first-line treatment but did not report baseline characteristics separately for this patient group [37].

The Japanese retrospective study by Tamada et al. (2018) included a total of 83 patients with mRCC who were treated with second-line axitinib or mTOR inhibitors (everolimus or temsirolimus) after first-line therapy with sunitinib [38]. The median (range) age was 68 years (41–84) for patients who received second-line axitinib and 63 years (43–77) for patients who received mTOR inhibitors [38]. Female patients were represented with 23.1% and 32.2% in the axitinib and mTOR study groups, respectively [38]. The proportion of patients in each IMDC risk group differed slightly between the axitinib and mTOR inhibitor cohorts at 21.2% and 32.3% in the favourable-risk groups, 55.8% and 45.2% in the intermediate-risk groups, and 21.2% and 22.6% in the poor-risk groups, respectively [38]. Most of the patients in both groups had ccRCC, with 96.2% in the axitinib group and 87.1% in the mTOR inhibitor group (Table S1) [38].

### 3.3. Summary of Evidence from Clinical and Observational Studies

#### 3.3.1. Progression-Free Survival

Table 3 summarises the PFS results from the identified studies. Axitinib demonstrated a statistically significant benefit in PFS compared with sorafenib in the AXIS trial, irrespective of the type of first-line therapy used [24,28]. In patients with prior sunitinib treatment, the median (95% CI) PFS with axitinib (N = 194) was 6.5 months (5.7–7.9) versus 4.4 months (2.9–4.7) with sorafenib (N = 195) (hazard ratio [HR]: 0.72; 95% CI: 0.57–0.90; *p* = 0.0022) [28]. In the patient groups with prior cytokine therapy, the median PFS with axitinib (N = 126) and with sorafenib (N = 125) was 12.2 months (10.2–15.5) and 8.2 months (6.6–9.5), respectively (HR: 0.505; 95% CI: 0.37–0.68; *p* < 0.0001) [28]. In the ESCAPE RCT, the median PFS with axitinib was 3.7 months (0.0–8.2) in patients who received prior sunitinib (N = 5) and 14.7 months (2.5–26.9) in patients with prior cytokine treatment (N = 13) (HR: 0.60; 95% CI: 0.20–1.79; *p* = 0.355) [29].

In the single-arm clinical trials, the median PFS of axitinib ranged from 7.1 to 15.7 months. Rixe et al. (2007) [32] observed a median PFS of 15.7 months (8.4–23.4) for axitinib, and the Japanese single-arm trial by Eto et al. (2014) [30] reported a median PFS of 11 months (9.2–12.0). In both studies, patients received axitinib after first-line cytokine treatment [30,32]. Rini et al. (2009) reported a median PFS of 7.1 months (3.9–7.6) in the subgroup of patients who received third-line axitinib after the failure of sorafenib and sunitinib (N = 14) [31]. In patients who received sorafenib and cytokines prior to axitinib treatment (N = 29), a median PFS of 9.1 months (7.1–21.4) was observed [31].

The study by Cesas et al. (2023) analysed PFS outcomes of second-line axitinib, cabozantinib and nivolumab stratified by IMDC risk groups after the failure of prior therapy with sunitinib [33]. Among these treatment sequences, the longest median second-line PFS (PFS2) was observed with sunitinib followed by axitinib (n = 17) in the IMDC favourable-risk group with 33.18 months (not reported [NR]) [33]. In patients who received cabozantinib (n = 4) and nivolumab (n = 5), the median PFS2 in the favourable-risk group was 27.15 months (NR) and 24.50 months (NR), respectively [33]. The difference in PFS2 between axitinib, cabozantinib, and nivolumab was not statistically significant [33]. In the intermediate-risk groups, the longest median PFS2 was observed in patients who received nivolumab (n = 25) at 27.76 months (NR) compared with 23.19 months (NR) and 21.55 months (NR) with axitinib (n = 30) and cabozantinib (n = 20), respectively [33]. In the small group of patients with a poor IMDC risk profile, median PFS2 was 12.05 months (NR) not reached and 11.26 months (NR) in the axitinib (n = 5), cabozantinib (n = 1) and nivolumab (n = 7) groups, respectively [33].

In the study by Tamada et al. (2018), PFS outcomes were compared between patients who received first-line sunitinib followed by second-line axitinib (n = 52) or mTOR inhibitors (n = 31) [38]. The study authors observed a significantly longer median PFS with axitinib of 8.7 months (NR) compared with mTOR inhibitors at 3.4 months (NR; *p* = 0.001) [38].

In the 148 patients with mRCC in the study by Facchini et al. (2019), the median PFS of axitinib after prior sunitinib was 7.14 months (5.78–8.5) [34]. The study authors analysed PFS according to several risk factors; patients who received a dose escalation of axitinib to 7 mg or 10 mg twice daily (n = 35) had a longer median PFS compared with those patients whose dose was not titrated (9.9 months [6.2–13.5] versus 6.4 months [5.78–8.5]; *p* = 0.1) [34].

Iacovelli et al. (2018) compared the median PFS between patients who received axitinib (n = 103) and everolimus (n = 79) after the failure of first-line treatment with sunitinib [36]. The median PFS was longer in patients who received axitinib with 5.5 months (4.3–6.7) versus patients who received everolimus with 4.6 months (2.6–6.5); the difference between the two treatment groups was not statistically significant (*p* = 0.7) [36].

#### 3.3.2. Overall Survival

The OS results from the identified studies are provided in Table 4. Median (95% CI) OS in the AXIS trial after first-line sunitinib was 15.2 months (12.8–18.3) and 16.5 months (13.7–19.2) with axitinib and sorafenib, respectively (HR: 0.997; 95% CI: 0.782–1.270; *p* = 0.4902) [31]. For patients who received cytokines as prior therapy, median OS was 29.4 months (24.5, not reported) and 27.8 months (23.1–34.5) with axitinib and sorafenib, respectively (HR: 0.81; 95% CI: 0.555–1.191; *p* = 0.1435) [31]. The difference in median OS between axitinib and sorafenib was not statistically significant [31]. For the ESCAPE trial, OS data were not reported [29].

In the single-arm clinical trials, the longest median OS of axitinib was 37.3 months (28.6–49.9) observed in Japanese patients who failed prior therapy with cytokines [30]. The second longest median OS was reported by Rixe et al. (2007) with 29.9 months (20.3, not reached) [32]. The shortest median OS outcomes were reported in the study by Rini et al. (2009) in patients who received third-line axitinib after prior lines of either cytokines and sorafenib (N = 29) or sunitinib and sorafenib (N = 14) with 18.5 months (8.4, not reached) and 11.5 months (7.1–15.9), respectively [31].

From the observational studies, the longest median OS of 69.5 months (NR) was reported in the study by Tamada et al. (2018) in Japanese patients who received first-line sunitinib followed by second-line axitinib (n = 52) [38]. The median OS with axitinib was statistically significantly longer compared with the study group of patients who received mTOR inhibitors (n = 31) after first-line sunitinib (33.4 months [NR]; *p* = 0.034) [38].

Géczi et al. (2020) compared OS outcomes in patients who received first-line sunitinib followed by second-line treatment with either axitinib (n = 128) or everolimus (n = 318) [35]. In this study, the median OS was also significantly longer in the sunitinib–axitinib group at 41.0 months (NR) compared with sunitinib, followed by everolimus at 21.7 months (NR; HR: 0.55 [0.42–0.72], *p* < 0.0001) [35].

Similarly, Iacovelli et al. (2018) analysed OS in patients treated with axitinib (n = 103) or everolimus (n = 79) after first-line therapy with sunitinib; however, in this study, the authors did not find a statistically significant difference between the two study groups [36]. The median OS with second-line axitinib and everolimus was 12.0 months (7.9–16.2) and 13.9 months (10.4–17.4), respectively (*p* = 0.3) [36].

Kang et al. (2023) compared survival outcomes of different first- and second-line treatment sequences, including sunitinib followed by axitinib and sunitinib followed by cabozantinib [37]. Whilst longer OS was observed with second-line axitinib versus cabozantinib, the difference was not statistically significant (HR: 0.795; *p* = 0.1773) [37]. The study authors also reported five-year OS rates that were higher with sunitinib followed by axitinib (51.44%) compared with sunitinib followed by cabozantinib (43.59%) [37].

In the Italian SAX study, the median OS for second-line axitinib after first-line sunitinib was 15.5 months (11–20.04) [34]. In those patients who had dose escalations to either 7 mg or 10 mg twice daily (n = 35), the median OS was 19.0 months (15.3–22.7) compared with 14.1 months (9.8–18.3) in patients without dose titration (n = 134) [34]. The difference between these two groups was not statistically significant (*p* = 0.115) [34].

#### 3.3.3. Response Rates

The response rates reported across the different studies are summarised in Table 5. In the AXIS trial, an objective response was achieved by 22 out of 194 patients (11.3%) receiving axitinib after prior sunitinib compared with 15 out of 195 patients (7.7%) in the sorafenib group; the difference was not statistically significant [27]. In the patient group treated with prior cytokine therapy, a statistically significant difference in ORR was observed with axitinib versus sorafenib; in the axitinib treatment arm, 41 out of 126 patients (32.5%) achieved an objective response versus 17 out of 125 patients (13.6%) in the sorafenib arm (*p* = 0.0002) [27]. Overall, no patient achieved a complete response in the AXIS trial [24,27,28]. In the ESCAPE RCT, 8 out of 13 patients (62%) who received cytokine therapy prior to axitinib achieved an objective response; of these, one patient achieved a complete response, and the other seven patients had a partial response to axitinib treatment [29]. None of the five patients who received prior sunitinib achieved an objective response [29].

From the single-arm trials, the highest ORR was observed in the Japanese study by Eto et al. (2014), with 52.6% (33 out of 64 patients) in patients who received second-line axitinib after the failure of cytokine therapy; all these patients had a partial response [30]. Rixe et al. (2007) reported an ORR of 44.2% (23 out of 52 patients) in patients who received axitinib after the failure of cytokine therapy; two of these patients achieved a complete response, and 21 patients had a partial response [32]. In the study by Rini et al. (2009), ORRs of 27.6% (8 out of 29 patients) and 7.1% (1 out of 14 patients) were reported for the subgroups of patients who received sorafenib and cytokines and sorafenib and sunitinib prior to axitinib, respectively [31].

Response rates to axitinib treatment after the failure of prior therapy with sunitinib or cytokines were reported by only one observational study, i.e., the SAX study from Italy by D’Aniello et al. (2016) [39] and Facchini et al. (2019) [34]. The authors reported an ORR of 16.6% (25 out of 148 patients) in patients with mRCC who received second-line axitinib after the failure of prior sunitinib [34]. One patient in this study achieved a complete response [34].

#### 3.3.4. Safety—Treatment Discontinuation Due to Adverse Events

In the subgroup of patients who received first-line sunitinib in the AXIS trial, fewer patients in the axitinib versus sorafenib arm discontinued treatment due to AEs, with 24 out of 192 patients (12.5%) and 37 out of 190 patients (19.5%), respectively [25]. Data on discontinuation due to AEs were not reported for the subgroup of patients who received first-line treatment with cytokines. In the ESCAPE trial, data on axitinib treatment discontinuation due to AEs were not reported [29].

In the single-arm studies by Rixe et al. (2007) [32] and Eto et al. (2014) [30], discontinuation due to AEs was reported in 19% (10 out of 52) and 25% (16 out of 64), respectively. In both studies, mRCC patients received first-line treatment with cytokines and second-line treatment with axitinib [30,32]. In the study by Rini et al. (2009), discontinuation rates were not reported separately for the subgroup of patients who received prior sunitinib or cytokines [31].

Safety data were reported in only two of the identified observational studies. The study by Tamada et al. (2018) did not report the rate of treatment discontinuation due to AEs but provided the type of AEs that led to treatment discontinuation [38]. In the axitinib group (n = 52), patients discontinued treatment due to gastrointestinal perforation, renal dysfunction, perianal abscess, diarrhoea, hyponatremia, and hoarseness, whereas patients discontinued treatment due to dermatitis, stomatitis, and skin rash in the mTOR inhibitor group (n = 31) [38].

Facchini et al. (2019) reported AEs associated with axitinib in those patients who received the standard dose of axitinib versus those who received an escalated dose of axitinib, and the occurrence of AEs was similar across the two groups [34]. Data on treatment discontinuation due to AEs were not reported.

#### 3.3.5. Dose Reductions and Dose Escalations

In the AXIS trial, dose reductions occurred in 121 patients (34%) and 192 patients (54%) in the overall axitinib and sorafenib groups, respectively [28]. In a total of 136 patients (38%), the daily axitinib dose was increased to more than 5 mg twice daily [28]. Data on dose reductions/escalations were not reported for the ESCAPE trial [29].

In the single-arm trial by Rixe et al. (2007), the axitinib dose was reduced in 15 patients (29%) [32]. Eto et al. (2014) reported that 46 patients (72%) had a reduction in the axitinib dose; in six patients (9%), the axitinib dose was increased to 7 mg (n = 5) and 10 mg (n = 1) twice daily, respectively [30]. In the publication by Rini et al. (2009), any dose modifications were not reported separately for the subgroup of patients who received sunitinib or cytokines prior to axitinib [31].

In the observational SAX study published by Facchini et al. (2019), 35 out of 148 patients (24%) had axitinib dose reductions; another 35 patients (24%) received an increased dose of axitinib of either 7 mg or 10 mg twice daily [34]. All patients in the SAX study were treated with axitinib after the failure of prior treatment with sunitinib [34].

#### 3.3.6. Health-Related Quality of Life

In the AXIS trial, HRQoL outcomes were assessed via the Functional Assessment of Cancer Therapy (FACT) Kidney Cancer Symptom Index (FKSI) and the EQ-5D [26]. For both instruments, scores remained stable during treatment with axitinib and sorafenib but declined with the occurrence of disease progression [26]. There was no statistically significant difference between the two treatment groups [26]. HRQoL data were not reported for the ESCAPE trial [29].

Of the single-arm trials, only the study by Rixe et al. (2007) assessed HRQoL in patients who received axitinib after the failure of cytokine therapy [32]. In this study, exploratory HRQoL analyses were conducted based on the patient-reported outcomes of the European Organisation for Research and Treatment of Cancer Quality of Life Questionnaire Core 30 (EORTC QLQ-C30) [32]. The study authors compared HRQoL between responders and non-responders to axitinib treatment and concluded that responders seemed to have better HRQoL outcomes compared with non-responders [32].

None of the identified observational studies reported data on HRQoL.

### 3.4. Study Quality Appraisal

Two RCTs were identified in this SLR, the AXIS and ESCAPE trials. The AXIS trial was of good quality; however, the potential risk of performance and detection bias remains due to the open-label design of the study. The quality of the ESCAPE trial was mixed as information on the concealment of the treatment allocation was missing, an open-label design was used, and an analysis set other than ITT was used. The quality of the non-randomised and observational studies was mixed. Whilst three studies exceeded 70% of the possible maximum score of the modified Downs and Black checklist, the score of the six remaining studies ranged between 52% and 68%.

## 4. Discussion

This SLR provides a comprehensive and transparent overview of relevant evidence around axitinib after the failure of prior cytokine and sunitinib treatment in aRCC. A total of 1817 records were screened from databases and conference websites, resulting in the inclusion of 210 publications. Given the focus of this manuscript on axitinib following treatment with sunitinib or cytokines, 16 publications were further assessed for efficacy and safety.

The AXIS trial demonstrated the superiority of axitinib over sorafenib in terms of PFS, ORR, and safety [24,27,28]. The median PFS with axitinib ranged from 6.5 months after prior treatment with sunitinib to 12.2 months after prior therapy with cytokines [28]. A slightly higher range of median PFS was observed in the single-arm clinical trials (7.1–15.7 months) [31,32]. When considering real-world evidence, prior observations from interventional studies were confirmed. The median PFS for patients receiving axitinib after prior therapy with either sunitinib or cytokines ranged between 5.5 months [36] and 8.7 months [38].

In terms of OS, the AXIS trial did not establish a statistically significant clinical benefit of axitinib compared with sorafenib [28]. The median OS with axitinib ranged from 15.2 months after prior treatment with sunitinib to 29.4 months after prior cytokine therapy [28]. OS outcomes were similar in the single-arm clinical trials. In the observational studies, the median OS ranged from 12 months [36] to 41.0 and 69.5 months as reported by Géczi et al. (2020) [35] and Tamada et al. (2018) [38], respectively. Of note, the study by Tamada et al. (2018) should be considered with care as the large median OS seems to be substantially different from the results of the other observational evidence.

Most of the identified studies in this SLR reported higher ORRs for axitinib compared with the results of the AXIS trial, where the ORR with axitinib ranged between 11.3% in the treatment arm with prior sunitinib and 32.5% in the treatment arm with prior cytokines [27]. Considering all identified clinical trials, ORRs ranged from 7.1% [31] to 62% [29]. Only one of the observational studies (Facchini et al., 2019) reported response rates (ORR: 16.6%) [34].

In terms of safety, axitinib showed favourable results in the AXIS trial compared with sorafenib in the subgroup of patients who received first-line treatment with sunitinib based on discontinuations due to AEs (12.5% versus 19.5%) [25]. Discontinuation rates due to AEs were reported in only two of the single-arm clinical trials but not in any of the observational studies. In the single-arm clinical trials, the discontinuation rates of axitinib due to AEs were slightly higher compared with the results in the AXIS trial, with 19% in the trial by Rixe et al. (2007) [32] and 25% reported by Eto et al. (2014) [30].

The flexible dosing of axitinib allows for dose reductions and dose escalations as needed for the individual patient, and axitinib’s short half-life typically helps to resolve any toxicities more quickly. In the AXIS trial, dose reductions and dose escalations were reported in 34% and 38% of the overall axitinib treatment arm, respectively [28]. One of the single-arm clinical trials reported a similar rate of dose reductions at 29% [32], whilst another single-arm clinical trial reported dose reductions in 72% of patients [30]. In the latter study, 9% of the patients also received an escalated dose [30]. Only one observational study reported on dose modifications with axitinib. In this study, 24% of patients had dose reductions, and another 24% of study participants had dose escalations [34].

Data on HRQoL were reported by only one RCT and one single-arm trial. None of the observational studies reported HRQoL outcomes. Overall, the evidence and assessment tools varied across studies, highlighting the complexity of assessing and comparing these outcomes. Furthermore, the limited number of studies underlines a paucity of HRQoL data on axitinib in aRCC and highlights the need for further research on HRQoL through observational studies.

This SLR has several strengths. Firstly, an extensive list of comparators was compiled based on clinical expert opinion and on recommended and approved treatments. Secondly, we gathered real-world evidence that complements the findings from interventional studies and allows for a more realistic view of treatment outcomes. Additionally, we utilised diverse information sources, including various databases and conferences, to ensure broad publication coverage and to include the latest findings. By following established guidelines from PRISMA 2020 and Cochrane, and with all records screened by two independent reviewers, methodological rigour and consistency were maintained, enhancing the credibility of our results. To our knowledge, this is the first SLR in the past 10 years evaluating therapies in aRCC considering both interventional and real-world evidence.

A limitation of this SLR is that comparisons between studies were challenging due to heterogeneity in the study and patient characteristics. Moreover, the comparison of axitinib to other treatments was limited given the small amount of comparative evidence from interventional and observational studies. Most of the latter compared axitinib with everolimus, but other treatment comparisons were sparse. Furthermore, the quality of the identified studies was mixed. Whilst the AXIS trial was of good quality, many other studies did not demonstrate that robust and unbiased methods were used. Finally, a general limitation of SLRs is the risk that not all available evidence has been captured, despite best efforts. Potentially, relevant evidence may have been presented at conferences that were not captured in this SLR or in manuscripts that may not have been identified by our search strategy.

In this manuscript, the presented data centred around the efficacy and safety outcomes of the label-conforming use of axitinib. However, a substantial body of evidence was also identified for studies assessing axitinib following treatments other than cytokines or sunitinib. Therefore, synthesising the evidence on axitinib’s off-label use presents an interesting opportunity for future research.

## 5. Conclusions

This SLR identified a large amount of data on axitinib in the treatment of aRCC. Axitinib is commonly used in clinical practice and has a well-characterised safety and efficacy profile in the treatment of patients with aRCC after the failure of prior treatment with sunitinib or cytokines. Moreover, evidence from observational studies shows that outcomes with axitinib observed in clinical trial settings can be replicated and even improved upon in real-world clinical practice.

## Figures and Tables

**Table 1:** PICOS criteria for the SLR.

PICOS	Inclusion Criteria	Exclusion Criteria
Population	Adult patients (≥18 years of age) with aRCC who have failed prior treatment with cytokines, TKI monotherapy, TKI-ICI combination, ICI monotherapy, or ICI combination therapy. Patients receiving second-line, third-line, or later lines of therapy.	Adult patients with aRCC receiving first-line treatment
Interventions/ Comparators	Axitinib Cabozantinib Everolimus Lenvatinib + everolimus Nivolumab Pazopanib Sunitinib Tivozanib Belzutifan	Interventions not listed in the inclusion criteria
Outcomes	Efficacy: OS (median, landmark time-point analysis) PFS, PFS2 (median, landmark time-point analysis) Response: OR, CR, PR, duration of response Duration of treatment Time to next therapy Safety: Incidence of any grade AE Incidence of grade 3–4 AE Incidence of specific AE Incidence of discontinuation due to AE Dose intensity Incidence of dose reduction HRQoL: Disease-specific and general	Outcomes not listed in the inclusion criteria
Study design	Randomised controlled trials Non-randomised interventional trials Pragmatic clinical trials (randomised or non-randomised) Observational or any RWE study design	Narrative reviews Prognostic studies Case reports Commentaries and letters Consensus reports Pooled analyses, SLRs, and meta-analyses ^1^
Other criteria	English language only Sample size > 20 participants for non-randomised interventional trials and observational studies	

^1^ Reference lists of pooled analyses, SLRs, and meta-analyses were reviewed to identify relevant studies. AE, adverse event; aRCC, advanced renal cell carcinoma; CR, complete response; HRQoL, health-related quality of life; ICI, immune checkpoint inhibitor; OR, overall response; OS, overall survival; PFS, progression-free survival; PFS2, second-line progression-free survival; PICOS, population, interventions/comparators, outcomes, study design; PR, partial response; RWE, real-world evidence; SLR, systematic literature review; TKI, tyrosine kinase inhibitor.

**Table 3:** Progression-free survival.

Author, Year	Median Follow-Up	Prior Treatment	Study Treatment	N	Median PFS (95% CI)
Randomised controlled trials					
Motzer, 2013 [28] AXIS (NCT00678392)	NR	1L sunitinib	2L axitinib	194	6.5 months (5.7–7.9)
			2L sorafenib	195	4.4 months (2.9–4.7)
		1L cytokines	2L axitinib	126	12.2 months (10.2–15.5)
			2L sorafenib	125	8.2 months (6.6–9.5)
Kadono, 2023 [29] ESCAPE (UMIN000012522)		1L sunitinib	2L axitinib	5	3.7 months (0.0–8.2)
	3 years	1L cytokines	2L axitinib	13	14.7 months (2.5–26.9)
Single-arm clinical trials					
Eto, 2014 [30] (NCT00569946)	NR	1L or 2L cytokines	≥2L axitinib	64	11 months (9.2–12.0)
Rini, 2009 [31] (NCT00282048)	22.7 months	Sunitinib + sorafenib	3L axitinib	14	7.1 months (3.9–7.6)
		Cytokines + sorafenib	3L axitinib	29	9.1 months (7.1–21.4)
Rixe, 2007 [32]	31 months	1L cytokines	2L axitinib	52	15.7 months (8.4–23.4)
Observational studies					
Cesas, 2023 [33]	29.26 months	1L sunitinib	IMCD risk favourable:		PFS2
			2L cabozantinib	4	27.15 months
			2L nivolumab	5	24.5 months
			2L axitinib	17	33.18 months
			IMCD risk intermediate:		
			2L cabozantinib	20	21.55 months
			2L nivolumab	25	27.76 months
			2L axitinib	30	23.19 months
			IMDC risk poor:		
			2L cabozantinib	1	Not reached
			2L nivolumab	7	11.26 months
			2L axitinib	5	12.05 months
Facchini, 2019 [34]	NR	1L sunitinib	2L axitinib	148	7.14 months (5.78–8.5)
			2L axitinib—patients with dose titration to 7 mg or 10 mg twice daily	35	9.9 months (6.2–13.5)
			2L axitinib—patients without dose titration	113	6.4 months (5.2–7.6)
Iacovelli, 2018 [36]	50.2 months	1L sunitinib	2L axitinib	103	5.5 months (4.3–6.7)
			2L everolimus	79	4.6 months (2.6–6.5), *p* = 0.7
Tamada, 2018 [38]	NR	1L sunitinib	2L axitinib	51	8.7 months
			2L everolimus or temsirolimus	31	3.4 months, *p* = 0.001

≥2L, second line and later lines; 1L, first line; 2L, second line; CI, confidence interval; IMDC, International Metastatic Renal Cell Carcinoma Database Consortium; NR, not reported; PFS, progression-free survival.

**Table 4:** Overall survival.

Author, Year	Median Follow-Up	Prior Treatment	Study Treatment	N	Median OS (95% CI)	Landmark OS
Randomised controlled trials						
Motzer 2013 [28] AXIS (NCT00678392)	NR	1L sunitinib	2L axitinib	194	15.2 months (12.8–18.3)	NR
			2L sorafenib	195	16.5 months (13.7–19.2)	NR
		1L cytokines	2L axitinib	126	29.4 months (24.5-NR)	NR
			2L sorafenib	125	27.8 months (23.1–34.5)	NR
Single-arm clinical trials						
Eto, 2014 [30] (NCT00569946)	NR	1L or 2L cytokines	≥2L axitinib	64	11 months (9.2–12.0)	NR
Rini, 2009 [31] (NCT00282048)	22.7 months	Sunitinib + sorafenib	≥2L axitinib	14	11.5 months (7.1–15.9)	NR
		Cytokines + sorafenib	≥2L axitinib	29	18.5 months (8.4-NR)	NR
Rixe, 2007 [32] (NCT00076011)	31 months	1L cytokines	2L axitinib	52	29.9 months (20.3-NR)	1-year: 78.8%
Observational studies						
Facchini, 2019 [34,39]	NR	1L sunitinib	2L axitinib	148	15.5 months (11–20)	NR
			2L axitinib with titrated dose	35	19 months (15.3–22.7), *p* = 0.115	NR
			2L axitinib with standard dose	113	14.1 months (9.8–18.3)	NR
Géczi, 2020 [35]	NR	1L sunitinib	2L axitinib	128	41 months	NR
			2L everolimus	318	21.7 months, *p* < 0.0001	NR
Iacovelli, 2018 [36]	50.2 months	1L sunitinib	2L axitinib	103	12 months (7.9–16.2)	NR
			2L everolimus	79	13.9 months (10.4–17.4), *p* = 0.3	NR
Kang, 2023 [37]	25 months	1L sunitinib	2L axitinib	300	HR: 0.795 (0.569–1.110), *p* = 0.1773	5-year: 51.44%
			2L cabozantinib	124	Reference	5-year: 43.59%
Tamada, 2018 [38]	NR	1L sunitinib	2L axitinib	51	69.5 months, *p* = 0.034	NR
			2L everolimus or temsirolimus	31	33.5 months	NR

≥2L, second line and later lines; 1L, first line; 2L, second line; CI, confidence interval; HR, hazard ratio; NR, not reported; OS, overall survival.

**Table 5:** Response rates.

Author, Year	Median Follow-Up	Prior Treatment	Study Treatment	N	Definition of Response	Response Outcome
Randomised controlled trials						
Ueda 2013 [27] AXIS (NCT00678392)	NR	1L sunitinib	2L axitinib	194	ORR	11.3%
			2L sorafenib	195	ORR	7.7%, *p* = 0.1085
			2L axitinib	194	CR	0%
			2L sorafenib	195	CR	0%
			2L axitinib	194	PR	11.3%
			2L sorafenib	195	PR	7.7%
		1L cytokines	2L axitinib	126	ORR	32.5%, *p* = 0.0002
			2L sorafenib	125	ORR	13.6%
			2L axitinib	126	CR	0%
			2L sorafenib	125	CR	0%
			2L axitinib	126	PR	32.5%
			2L sorafenib	125	PR	13.6%
Kadono, 2023 [29] ESCAPE (UMIN000012522)	3 years	1L cytokines	2L axitinib	13	ORR	62%
					CR	8%
					PR	54%
		1L sunitinib	2L axitinib	5	ORR	0%
					CR	0%
					PR	0%
Single-arm clinical trials						
Eto, 2014 [30] (NCT00569946)	NR	1L or 2L cytokines	≥2L axitinib	64	ORR	51.6%
					CR	0%
					PR	51.6%
					SD	43.8%
Rini, 2009 [31] (NCT00282048)	22.7 months	Sunitinib + sorafenib	≥2L axitinib	14	ORR	7.1%
		Cytokines + sorafenib	≥2L axitinib	29	ORR	27.6
Rixe, 2007 [32] (NCT00076011)	31 months	1L cytokines	2L axitinib	52	ORR	52.6%
					CR	4%
					PR	28%
					SD	42%
Observational studies						
Facchini, 2019 [34,39]	NR	1L sunitinib	2L axitinib	148	ORR	16.6%
					CR	0.6%
					PR	16%

≥2L, second-line and later lines; 1L, first-line; 2L, second-line; CR, complete response; NR, not reported; ORR, overall response rate; PR, partial response; SD, stable disease.

## Supplementary Materials

The following supporting information can be downloaded at: https://www.mdpi.com/article/10.3390/cancers16152706/s1, Table S1: Baseline characteristics of patients with aRCC who received treatment with sunitinib/cytokines prior to axitinib. References [29,30,32,33,34,35,36,38] are cited in the supplementary materials.
