# Supplementary material for: Axitinib after Treatment Failure with Sunitinib or Cytokines in Advanced Renal Cell Carcinoma—Systematic Literature Review of Clinical and Real-World Evidence
Source: Cancers (Basel). 2024 Jul 30;16(15):2706. doi: 10.3390/cancers16152706 (PMC11312084; doi:10.3390/cancers16152706)
Supplement: Supplementary file 1 [file cancers-16-02706-s001.zip › cancers-3093205-supplementary.pdf]

Supplementary Material:

Axitinib after treatment failure with sunitinib or cytokines in advanced renal cell carcinoma – systematic literature review of clinical and real-world evidence

Table S1. Baseline characteristics of patients with aRCC who received treatment with sunitinib/cytokines prior to axitinib*

Author, year	Treatment arm (n)	Prior treatment	Age (range)	Female patients	ECOG PS score	MSKCC score	IMDC Score	Metastatic disease	Histology	Prior nephrectomy
**RCTs**										
Kadono, 2023[1] ESCAPE (UMIN000012522)	2L axitinib (13)	1L interleukin-2 + interferon-alpha (54.5%)<br>1L sunitinib (45.5%)	NR	NR	NR	NR	NR	100%	NR	NA
**Single-arm clinical trials**										
Eto, 2014[2] (NCT00569946)	≥2L axitinib (64)	1L or 2L interleukin-2 and/ or interferon-alpha (100%)	Median: 63 (34-80)	20 (31%)	0: 89%<br>1: 11%	F: 16%; I: 77%; P: 7%	NR	100%	ccRCC: 97%; Papillary cell: 2%	100%
Rixe, 2007[3] (NCT00076011)	2L axitinib (52)	1L cytokines (100%)<br>1L interferon alone (52%)<br>1L interleukin-2 alone (17%)<br>1L interferon and interleukin-2 (15%)<br>1L cytotoxic chemotherapy (15%)<br>1L previous radiotherapy (19%)	Median 59 (35-85)	12 (23%)	0: 60%<br>1: 40%	F: 42%; I or higher: 58%	NR	100%	ccRCC: 98%; Papillary cell: 2%	94%
**Observational studies**										
Cesas, 2023[4]	2L cabozantinib (25)	1L sunitinib (100%)	NR	NR	NR	NR	F: 16%; I: 80%; P: 4%	NR	NR	NR

Author, year	Treatment arm (n)	Prior treatment	Age (range)	Female patients	ECOG PS score	MSKCC score	IMDC Score	Metastatic disease	Histology	Prior nephrectomy
	2L nivolumab (38)						F: 13.5%; I: 67.6%; P: 18.9%			
	2L axitinib (58)						F: 32.7%; I: 57.7%; P: 9.6%			
	2L everolimus (2)						NR			
Facchini, 2019[5]	2L axitinib (148)	1L sunitinib (100%)	Median: 62 (35-85)	73 (49.3%)	0: 55.4% 1: 41.3% 2: 3.3%	F: 27.7%; I: 60.8%; P: 11.5%	F: 24.3%; I: 60.1%; P: 15.5%	100%	ccRCC: 94%	90.5%
Géczi, 2020[6]	2L axitinib (128)	1L sunitinib (100%)	Mean: 62.1	NR	NR	NR	NR	100%	NR	NR
	2L everolimus (318)		Mean: 60.9	NR	NR	NR	NR	100%	NR	NR
Iacovelli, 2018[7]	2L everolimus (79)	1L sunitinib (100%)	Median: 60.6	30 (38%)	NR	NR	F: 21.5%; I: 65.8%; P: 12.7%	100%	ccRCC: 100%	91%
	2L axitinib (103)		Median: 58.3	20 (19.4%)	NR	NR	F: 18%; I: 67%; P: 15%	100%	ccRCC: 100%	89.3%
Tamada, 2018[8]	2L axitinib (52)	1L sunitinib (100%)	Median: 68 (41-84)	12 (23.1%)	NR	F: 21.2%; I: 55.8%; P: 21.2%; Unknown: 1.9%	NR	100%	ccRCC: 96.2%	NA
	2L everolimus/ temsirolimus (31)		Median: 63 (43-77)	10 (32.3%)	NR	F: 32.3%; I: 45.2%; P: 22.6%	NR	100%	ccRCC: 87.1%	NA

* This table includes only those studies which provided data on patient populations/subgroups of patients who received sunitinib or cytokines prior to axitinib.
Abbreviations: 1L, first-line; 2L, second-line; ccRCC, clear-cell renal cell carcinoma; ECOG PS, Eastern Cooperative Oncology Group performance status; F, favourable; I, intermediate; IMDC, International Metastatic Renal Cell Carcinoma Database Consortium; MSKCC, Memorial Sloan Kettering Cancer Center; n, number of patients; NR, not reported; P, poor; RCT, randomised controlled trial

References:

1. Kadono Y.; Konaka H.; Nohara T.; et al. Efficacy and Safety of First-Line Cytokines Versus Sunitinib and Second-Line Axitinib for Patients with Metastatic Renal Cell Carcinoma (ESCAPE Study): A Phase III, Randomized, Sequential Open-Label Study. *Cancers*, **2023**, 15(10), 2745, doi:10.3390/cancers15102745.
2. Eto M.; Uemura H.; Tomita Y.; et al. Overall survival and final efficacy and safety results from a Japanese phase II study of axitinib in cytokine-refractory metastatic renal cell carcinoma. *Cancer Sci*, **2014**, *105(12)*, 1576-83, doi: 10.1111/cas.12546.
3. Rixe O.; Bukowski R.M.; Michaelson M.D.; et al. Axitinib treatment in patients with cytokine-refractory metastatic renal-cell cancer: a phase II study. *Lancet Oncol*, **2007**, *8(11)*, 975-84. doi: 10.1016/S1470-2045(07)70285-1.
4. Cesas A.; Urbonas V.; Tulyte S.; et al. Sequential treatment of metastatic renal cell carcinoma patients after first-line vascular endothelial growth factor targeted therapy in a real-world setting: epidemiologic, noninterventional, retrospective–prospective cohort multicentre study. *J Cancer Res Clin Oncol*, **2023**, *149(10)*, 6979-6988, doi:10.1007/s00432-023-04645-x.
5. Facchini G. ; Rossetti S.; Berretta M.; et al. Second line therapy with axitinib after only prior sunitinib in metastatic renal cell cancer: Italian multicenter real world SAX study final results. *J Transl Med*, **2019**, *17(1)*, 296, doi:10.1186/s12967-019-2047-4.
6. Géczi L, Bodoky G, Rokszin G, Fábián I, Torday L. Survival Benefits of Second-line Axitinib Versus Everolimus After First Line Sunitinib Treatment in Metastatic Renal Cell Carcinoma. *Pathol Oncol Res*, **2020**, *26(4)*, 2201-7, doi:10.1007/s12253-020-00809-z.
7. Iacovelli R.; Cossu Rocca M. ; Galli L.; et al. The outcome to axitinib or everolimus after sunitinib in metastatic renal cell carcinoma. *Anti-Cancer Drugs*, **2018**, *29(7)*, 705-709, doi:10.1097/CAD.0000000000000632.
8. Tamada S.; Iguchi T.; Kato M.; Yasuda S.;, Yamasaki T.; Nakatani T. Second-line treatment after sunitinib therapy in patients with renal cell carcinoma: a comparison of axitinib and mammalian target of rapamycin inhibitors. *Oncotarget*, **2018**, *9(97)*, 37017-37025, doi:10.18632/oncotarget.26439.
